# Challenges in the diagnosis of periodic fever, aphthous stomatitis, pharyngitis, and adenitis syndrome in developing countries—A decade of experience from North India

**DOI:** 10.3389/fimmu.2022.958473

**Published:** 2022-09-20

**Authors:** Aaqib Zaffar Banday, Vibhu Joshi, Kanika Arora, Rohit Sadanand, Suprit Basu, Rakesh Kumar Pilania, Ankur Kumar Jindal, Pandiarajan Vignesh, Anju Gupta, Saniya Sharma, Manpreet Dhaliwal, Amit Rawat, Surjit Singh, Deepti Suri

**Affiliations:** Allergy Immunology Unit, Department of Pediatrics, Advanced Pediatrics Centre, Post Graduate Institute of Medical Education and Research (PGIMER), Chandigarh, India

**Keywords:** adenitis, aphthous stomatitis, periodic fever, PFAPA, pharyngitis, thalidomide

## Abstract

**Background:**

Reports of periodic fever, aphthous stomatitis, pharyngitis, and adenitis (PFAPA) syndrome from developing countries are sparse. Recognizing PFAPA is often challenging in these regions due to a higher incidence of infectious illnesses and significant resource constraints. Herein, we present our experience from North India regarding the diagnosis and management of PFAPA syndrome.

**Methods:**

We reviewed cases of non-monogenic periodic fever syndrome diagnosed at our center from January 2011 to December 2021. A total of 17 children who fulfilled the Marshall criteria for PFAPA syndrome were included. Data regarding basic clinical features, treatment/outcome, and performance of the recently proposed Eurofever/PRINTO and Takeuchi criteria were analyzed.

**Results:**

Besides recurrent fever, the triad of oral aphthae, pharyngitis, and adenitis was noted in only 18% of patients. Episodes of exudative pharyngitis/tonsillitis were documented in 24%. These figures were lower than the values reported from developed countries. The Takeuchi and Eurofever/PRINTO criteria were fulfilled in 76% and 71% cases, respectively. In addition to antipyretics and supportive care, intermittent steroid therapy was the main treatment modality used. Additional treatment with colchicine (*n* = 3) and thalidomide (*n* = 1) was used successfully in a few patients. Before the diagnosis of PFAPA, all patients had received multiple courses of antimicrobials (without microbiological confirmation). These included multiple courses of antibacterials for fever, pharyngotonsillitis, and/or cervical adenitis in all patients and antivirals for fever and aphthous stomatitis in a patient. Empiric antitubercular therapy had also been administered in two patients.

**Conclusions:**

A significant proportion of patients with PFAPA seem to remain undiagnosed in the Indian subcontinent. Increased awareness and improvement in basic healthcare facilities are crucial in enhancing the recognition of PFAPA, which would eliminate the unprecedented scale of undesirable antimicrobial use in such children.

## Introduction

Periodic fever, aphthous stomatitis, pharyngitis, and adenitis (PFAPA) syndrome is one of the most common autoinflammatory syndromes in the pediatric population (1). Genetic susceptibility and risk factors associated with PFAPA are being increasingly evaluated; however, no causative monogenic etiology has been identified to date (1, 2). First reported by Marshall et al. in 1987, the diagnosis of PFAPA to date remains purely clinical. While various criteria have been developed over the past 35 years for its diagnosis, the clinical features described in the first report still form the mainstay of diagnosis (3). Marshall et al. later proposed diagnostic criteria for PFAPA in mid-1989 (4, 5). Recently, in 2019, the Pediatric Rheumatology International Trials Organisation (PRINTO) provided clinical classification criteria for prototype autoinflammatory diseases including PFAPA, which were based on Eurofever registry data (hereafter referred to as Eurofever/PRINTO criteria) (6). Although these “classification” criteria are not designed to diagnose PFAPA, they may serve as a useful resource in clinical practice. Also in 2019, Takeuchi et al. proffered novel diagnostic criteria that are based on data from Japanese patients with PFAPA (hereafter referred to as Takeuchi criteria) (7).

Most data on PFAPA syndrome are derived from developed countries (8). There is a significant paucity of relevant data from developing countries including the Indian subcontinent. Moreover, diagnosis of PFAPA in tropical developing countries is often complicated given the rampant incidence of infectious illnesses in the said regions (resulting in frequent episodes of fever and other manifestations). Additionally, given the constraints in microbiological diagnoses and lack of awareness regarding autoinflammatory syndromes, manifestations of PFAPA syndrome may be considered infectious illnesses (such as streptococcal pharyngitis/tonsillitis, tubercular lymphadenitis) and treated empirically in such resource-constraint settings. Furthermore, a suspicion of a primary immunodeficiency disease (PID), also termed inborn error of immunity (IEI), may also arise in patients with PFAPA due to recurrent episodes of fever (and other manifestations), which are often considered infectious illnesses and treated with antimicrobials. Herein, we present our single-center experience from North India regarding the diagnosis and management of PFAPA syndrome. We highlight the challenges faced in developing countries in diagnosing the said syndrome. As we note, many patients with PFAPA remain undiagnosed for many years and are treated with multiple courses of antimicrobials including prolonged therapy with anti-tubercular drugs. This study is among the few reports of PFAPA from the developing regions of the world.

## Methods

This hospital-based retrospective study was conducted at the Allergy Immunology Unit, Department of Pediatrics, Advanced Pediatric Center, Post Graduate Institute of Medical Education and Research (PGIMER), Chandigarh, North India. PGIMER is the largest tertiary-care referral center in the region dedicated to the diagnosis and treatment of autoinflammatory disorders and periodic fever syndromes in the pediatric population. We reviewed cases of periodic fever syndrome diagnosed and/or treated at our center from January 2011 to December 2021. A total of 26 patients with periodic fever syndrome who lacked the diagnosis of a monogenic autoinflammatory disease were included. After a detailed case review, 17 children who fulfilled the Marshall criteria (4) for PFAPA syndrome were included in the final analysis.

Data regarding basic clinicolaboratory features, treatment, and outcomes were entered in an Excel spreadsheet (Office Professional Plus 2010, Microsoft Corporation). In children >5 years, weight, height, and body mass index (BMI) *z*-scores at presentation were calculated according to the Indian Academy of Pediatrics (IAP) reference values, while in children ≤5 years at diagnosis, weight, height, and weight-for-height z-scores were calculated according to World Health Organization growth standards (9, 10). The Statistical Package for Social Sciences software (SPSS, version 22, IBM Corporation) was used for data analysis. Given the small sample size and for the sake of uniformity, all continuous data (irrespective of parametric or non-parametric distribution) have been expressed as median (25th percentile, 75th percentile). Values of range [minimum–maximum] have been provided wherever appropriate. In cases where data regarding a continuous variable is not available for the whole cohort of 17 patients, the number for which the said data is available is shown as (*n* = *x*). Discrete data have been expressed as percentages (% [a/b]), with “a” denoting the number of patients out of the total cohort “b” for which the said data were available. Lack of the fraction “a/b” adjacent to the value of percentage reflects that data pertain to the whole cohort of 17 patients. Percentages have been rounded to the nearest whole number for clarity/brevity as the number of participants in our study was much less than 100. Individual patient(s) with notable clinical profile(s) (especially uncustomary features) are referred to as {P_z_} where “z” indicates the patient number.

## Results

**Figure 1:**
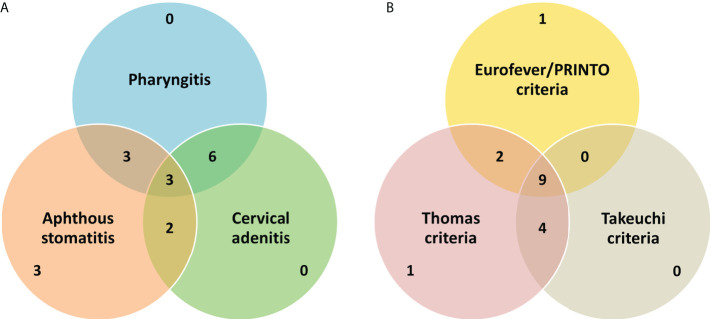
A brief summary of the basic clinical features **(A)** and performance of the various criteria for PFAPA **(B)** in our cohort. Values in the Venn diagrams refer to the absolute number of patients fulfilling particular diagnostic/classification criteria or having classical clinical manifestation(s).

We included 17 patients (47% female) with PFAPA syndrome in our study. Age at onset of symptoms was 2.10 (0.92, 3.00) years [1 month–6.33 years] and age at diagnosis was 3.55 (2.53, 6.76) [1.06–13.34] years. Only one patient {P_7_} had disease onset at >5 years. Interval between onset and diagnosis was 2.01 (0.90, 3.85) [0.39–7.35] years. Diagnosis was established within the first year of onset of symptoms in 29%. All patients with PFAPA had self-limiting episodes of febrile illness (subsequently referred to as attacks/flares) lasting 2–7 days. Periodicity of fever was noted in all patients with an asymptomatic intercurrent period (in between attacks). At presentation, the attacks had recurred at intervals of 3 to 6 weeks in 88% of patients. Recurrent episodes of pharyngitis/tonsillitis were noted in 71%; however, episodes of exudative pharyngitis/tonsillitis were documented in only 24% of patients. Recurring episodes of aphthous stomatitis and cervical adenitis were each noted in 65% (**Figure 1A**). Gastrointestinal symptoms of abdominal pain and vomiting were observed in 24% and 18%, respectively, while none of the patients had diarrhea during attacks. None of the patients had respiratory symptoms (including cough and chest pain) during attacks. Two patients provided history of intermittent episodes of rash with or without other features of PFAPA (including fever); however, none of the patients had a rash at presentation to us or during follow-up. None of the patients in our cohort had arthritis (before presentation or during follow-up). All included patients were of North Indian ancestry; 10 patients belonged to Hindu, 5 to Sikh, and 2 to Muslim families. Parental consanguinity was noted in one patient {P_2_} and none of the patients provided history of an illness similar to PFAPA. All patients had normal anthropometry at diagnosis of PFAPA (weight, height, and weight-for-height/BMI *z*-scores > −2 according to age- and sex-specific references). Height and weight *z*-scores at diagnosis were −0.460 (−1.045, 0.224) and −0.489 (−1.370, −0.200), respectively.

On laboratory evaluation, all patients had elevated inflammatory parameters (white cell count or erythrocyte sedimentation rate) during PFAPA attacks. SAA levels were not measured in any patient or were serum IgD levels. Most of our patients (94%) fulfilled the Thomas criteria (11) for PFAPA (all except {P_7_} who had disease onset at >5 years). Only 71% of patients fulfilled the Eurofever/PRINTO classification criteria (6) for PFAPA. The Takeuchi diagnostic criteria (7) were fulfilled in 76% of cases. Only one patient did not fulfill both the Eurofever/PRINTO and Takeuchi criteria (the rest fulfilled either of the two). In addition to Marshall’s criteria, all patients fulfilled at least one of the other criteria (Thomas, Eurofever/PRINTO, or Takeuchi criteria) (**Figure 1B**).

In two (12%) patients, only paracetamol and supportive therapy were used during flares of PFAPA. Naproxen was used in one (6%) patient during attacks with a good response. In addition to antipyretics and supportive care, intermittent steroid (oral prednisolone) therapy was the main treatment modality used (during attacks) in the majority (82%) of patients. The dose employed most often was 1 mg/kg on day 1 of illness. A second dose of oral prednisolone was given on day 2 of the illness if fever persisted. Fever responded briskly to steroids in 86% [12/14] and a partial response was noted in the rest. Administration of corticosteroids resulted in a decreased time interval between flares in five patients. Additional treatment with oral colchicine was given in three (18%) patients. Reasons for additional therapy included suboptimal response to steroids or increase in flare frequency. One of the two patients who showed partial response to steroids also responded poorly to colchicine therapy meriting the addition of oral thalidomide {P_10_}. Notably, this patient had a very young age (1 month) at the onset of symptoms reminiscent of PFAPA. Adenotonsillectomy was not performed in any patient in our cohort. Also, anti-IL-1 therapy was not utilized in any of our patients due to availability constraints.

Before the diagnosis of PFAPA, all patients had received multiple courses of antimicrobials. These included multiple courses of antibacterials for fever, pharyngotonsillitis, and/or cervical adenitis in all patients and (one course of) oral antivirals for fever and aphthous stomatitis in one (6%) patient. None of these patients had a microbiological diagnosis of an infection (e.g., for pharyngotonsillitis, aphthous stomatitis). Workup for tuberculosis had been performed in 71%, which was negative in all patients except two patients with Mantoux positivity. Empiric antitubercular therapy had been administered for recurrent episodes of fever in two (12%) patients including one patient with Mantoux positivity and cervical adenitis (presumed tubercular lymphadenitis). Both of these patients did not respond to antitubercular therapy and were subsequently treated for PFAPA. Immunoglobulin (IgG, IgA, and IgM) levels had been performed in 65% of patients (often due to a suspicion of an IEI), which were normal in all according to age- and sex-specific cutoffs.

Commercial targeted next-generation sequencing (NGS) for monogenic autoinflammatory diseases or whole-exome sequencing was performed in five (29%) patients. No relevant pathogenic or likely pathogenic variant was identified in any of these patients. NGS was performed in patients with young age (<6 months) at disease onset or those who required additional treatment besides steroids. NGS was not performed in the two patients with history of skin rash. Notably, no pathogenic or likely pathogenic variant was identified in the child with parental consanguinity and partial response to steroids {P_2_}. The targeted NGS panel, employed in two of the five patients, includes >40 genes known to cause autoinflammatory diseases including type I interferonopathies (e.g., *ADA2*, *TMEM173*, and *TREX1*), inflammasomopathies (e.g., *MEFV*, *MVK*, and *NLRP3*), and non-inflammasome-associated disorders (e.g., *PSTPIP1*, *TNFAIP3*, and *TNFRSF1A*).

Median duration of follow-up in our study was 3.43 years with a total follow-up duration of 68.62 patient-years. Improvement in fever spikes was noted with increasing age in 76% of our patients while the remaining continued to have PFAPA attacks. The clinical course was uneventful in all patients and no adverse sequels of PFAPA attacks were observed.

## Discussion

Although PFAPA is the commonest periodic fever syndrome in the pediatric population, reports from the developing regions of the world are sparse. Herein, we report our experience regarding the diagnosis and management of PFAPA spanning over a decade. This study is among the very few reports of PFAPA from the Indian subcontinent. We have also analyzed briefly the performance of the recently proposed classification and diagnostic criteria in our cohort.

Significant delay in the establishment of diagnosis was noted in our study. The median and the maximum time intervals to diagnosis were 2 and 7.3 years, respectively. Even in developed regions of the world, significant delays in diagnosis of PFAPA have been reported (12–14). In one of the largest multicentric cohort of patients with PFAPA from Europe (14), the median and the maximum duration of diagnostic delay were 2.3 and ≥20 years, respectively, suggesting that these children may continue to remain symptomatic till adulthood. One of the important reasons for delays in the establishment of diagnosis is poor recognition of this entity by healthcare providers. As PFAPA seems to be grossly underdiagnosed in children in developing nations, the delays in diagnosis may be higher than we have observed. The first report on PFAPA from Iran (15) reported a maximum diagnostic delay of up to 11 years. Nonetheless, diagnosis of PFAPA in developing countries is especially challenging given the often higher burden of infectious illnesses and presence of significant resource constraints.

Besides recurrent/periodic fever, the typical diagnostic triad (embodied in the name PFAPA) is usually seen in only a subset of patients. In the first description of PFAPA (Marshall et al.), one-third of patients had the complete triad while one-sixth had only one clinical manifestation (3). In our study, the triad was seen in only 18% of patients. The majority (65%) had two manifestations while the rest had only one clinical manifestation. Other reports from developing countries seem to suggest that the proportion of patients who fulfill all three major features may be lower than in developed countries. Mehregan et al. reported ~24% of Iranian patients with PFAPA to fulfill the triad (15). Studies on large cohorts of patients with PFAPA from developed regions have observed a higher proportion of patients with the clinical triad. Hofer et al. reported ~44% of the patients in their multicentric PFAPA cohort and Perko et al. reported that 53% of Slovenian patients with PFAPA manifested with the classical clinical triad (13, 14). The reasons for this discrepancy remain unclear; however, recall bias and less-meticulous documentation of PFAPA flares in resource-constraint settings (e.g., lack of digitized medical records) may play a crucial part. This hypothesis may be supported by the fact that the proportion of patients with exudative pharyngitis/tonsillitis in our study (24%) was lower compared to most of the previously published reports from developed regions. Similarly, exudative pharyngitis was reported in one-fourth of 16 patients with PFAPA in a single-center study from a countryside province in west Turkey (16). Conversely, exudative pharyngitis has been reported in ~38% of European patients with PFAPA (14), ~59% of Roman children with PFAPA (17), and in more than two-thirds of Japanese patients (7) with PFAPA. In an old report on 28 patients with PFAPA from Israel, Padeh et al. (18) incorporated exudative tonsillitis as an inclusion criterion for PFAPA (thereby seen in all patients studied). Whether the clinical profile of children/adolescents with PFAPA is different in tropical developing countries needs further evaluation. However, this seems unlikely given the presence of significant confounding factors in the said resource-constraint settings.

The performance of the recently proposed Eurofever/PRINTO and Takeuchi criteria in developing countries has not been assessed so far. In a large single-center cohort of 322 patients with PFAPA from Istanbul, Turkey, ~90% of patients fulfilled the newly proposed Takeuchi diagnostic criteria (for PFAPA) (19). In our cohort, about three-fourths of patients fulfilled the said criteria. Lack of similar family history, unavailability of serum IgD measurements, and lower frequency of documented exudative pharyngitis are the likely reasons for a lower proportion of PFAPA patients fulfilling the Takeuchi criteria. As noted previously, exudative pharyngitis/tonsillitis, presence of family history, and elevated IgD are separate surrogate criteria (in the Takeuchi criteria) while other typical clinical manifestations of PFAPA are grouped under a single surrogate criterion (7). The Eurofever/PRINTO classification criteria were fulfilled by 71% of patients of in our study. In the validation cohort of the Eurofever/PRINTO classification criteria, the sensitivity of PFAPA criteria was 66%; however, as these criteria were designed to “classify” PFAPA, the specificity was 97% (6). Given the under-recognition of PFAPA in developing countries, it may be preferable to utilize criteria with the highest sensitivity for diagnosis of PFAPA (although at the cost of optimal specificity). However, employing such an approach may not be feasible in developing regions where familial Mediterranean fever may be prevalent.

Similar to previously published reports, brisk response to steroids was noted in the majority of patients in our study (8, 20). Colchicine was used in three patients with good response in two. Nearly half to two-thirds of patients with PFAPA have been reported to respond to colchicine, which is comparable to our findings (8). Notably, we used thalidomide in one patient who had a poor response to steroids and colchicine. Thalidomide has been used previously in two adults with PFAPA syndrome with good response (21, 22). One of these patients had shown a partial response to colchicine (22) as we have also noted. PFAPA syndrome is associated with elevated IL-1/IL-18, and IFN-γ gene signatures (23). Anti-IL-1 agents (anakinra, canakinumab, and rilonacept) are not currently marketed in India. These biologics, although very expensive, can be procured on “named patient basis” from international pharmacies. Non-availability of anti-IL-1 agents prompted the use of thalidomide in one of our patients. Efficacy of thalidomide in PFAPA may arise from the inhibition of caspase-1, which converts pro-IL-1β and pro-IL-18 to biologically active IL-1β and IL-18, respectively (24, 25). Moreover, thalidomide may also inhibit the lymphocytic production of IFN-γ (26–28). Ours is among the very few reports describing the effectiveness of thalidomide in pediatric PFAPA. In addition to prophylactic therapy with colchicine or cimetidine, thalidomide prophylaxis may also be a feasible option in PFAPA; however, the lack of ample data precludes definite conclusions. Currently, thalidomide prophylaxis is not included in the Childhood Arthritis and Rheumatology Research Alliance (CARRA) consensus treatment plans for PFAPA (29). Also, occurrence of non-serious (drowsiness, fatigue, and constipation) and serious adverse events (neuropathy, venous thromboembolism, and hypersensitivity reactions) may complicate the use of thalidomide in pediatric clinical practice (30). Thalidomide needs to be utilized with extreme caution in adolescent female patients given the high risk of teratogenicity (including abortions) and propensity to cause amenorrhea (30).

We noted the rampant use of (multiple courses of) antimicrobials even without a microbiological diagnosis in patients later diagnosed with PFAPA. Other studies on PFAPA from developing countries have also reported the use of antimicrobials in >80% of patients with PFAPA (15). Few patients in our cohort also received many months of antitubercular therapy adding to the physical and psychological morbidity in patients with PFAPA (31). Given the lack of universal health insurance, injudicious use of antibacterials translated to an increased financial burden faced by caregivers of children with PFAPA. Increased awareness is imperative to ensure timely diagnosis and prevent overuse of antimicrobials, especially in settings where infectious illnesses are common. Furthermore, improvement in basic healthcare facilities is vital, which would help attain a microbiological diagnosis for clinical manifestations mimicking PFAPA (e.g., point-of-care streptococcal antigen testing for pharyngotonsillitis).

Lastly, a large number of IEIs including autoinflammatory diseases remain unrecognized in resource-constraint settings. Genetic evaluation forms the mainstay of definitive diagnosis for monogenic IEIs (and autoinflammatory diseases) (32). Increasing availability of NGS in developing countries would enhance the recognition of these disorders. However, it is also important to prevent injudicious use of labor-intensive high-throughput sequencing technologies as the costs remain significant in the context of resource-constraint settings where universal health insurance is often lacking. In patients suspected to have PFAPA, careful utilization of NGS is prudent as no monogenic cause of this syndrome has been identified so far (1). Moreover, consensus on indications for NGS (targeted panel or whole exome/genome sequencing) testing in patients suspected to have PFAPA is lacking currently (33). Autosomal recessive forms of IEI are more common in our region given the higher rates of consanguinity and endogamous marriages; however, consanguinity was noted in only one patient with PFAPA in our study (34). Furthermore, recurrent episodes of pharyngitis (with or without exudates) are distinctly uncommon as a major disease-specific manifestation in the majority of patients with an immune deficiency disorder (35). These basic clinical features may help to distinguish IEI from PFAPA and facilitate a more targeted evaluation and management.

### Limitations

An important limitation of this study is the small number of patients with PFAPA included in our cohort. Moreover, genetic evaluation for monogenic autoinflammatory diseases was performed in five patients only. Notably, although recurrent episodes of aphthous stomatitis were seen in the majority, A20 haploinsufficiency (AD *TNFAIP3* LOF, which may also mimic PFAPA) could be excluded in these few patients only (36).

## Conclusions

Although PFAPA is being increasingly diagnosed in the Indian subcontinent, a significant proportion of patients with PFAPA seem to remain undiagnosed given the huge population of over 1.5 billion in this region. Increased awareness and improvement in basic healthcare facilities are crucial in enhancing the recognition of PFAPA, which would eliminate the unprecedented scale of undesirable antimicrobial use in such children.

## Data availability statement

The original contributions presented in the study are included in the article. Further inquiries can be directed to the corresponding author.

## Ethics statement

This study was approved by the Review Board, Department of Pediatrics, Advanced Pediatric Center, PGIMER, Chandigarh (No. 53-22). Informed consent was obtained from the legal guardians of the patients before inclusion in the study.

## Author contributions

AB: Writing of initial draft of manuscript, editing and revision of manuscript, literature search and review, data acquisition and analysis, and patient management. VJ, KA, and RS: Editing and revision of manuscript, literature search and review, data acquisition and analysis, and patient evaluation. SB, RP, AJ, PV, AG, SSh, MD, and AR: Editing and critical revision of manuscript, data acquisition, and patient evaluation and management. SSi/DS: Inception of idea, editing/revision of manuscript and overall supervision of literature review, data acquisition, and patient management. All authors approve the final version of the manuscript.

## Conflict of interest

The authors declare that the research was conducted in the absence of any commercial or financial relationships that could be construed as a potential conflict of interest.

## Publisher’s note

All claims expressed in this article are solely those of the authors and do not necessarily represent those of their affiliated organizations, or those of the publisher, the editors and the reviewers. Any product that may be evaluated in this article, or claim that may be made by its manufacturer, is not guaranteed or endorsed by the publisher.
